# Supplementary material for: Bridging cell-scale simulations and radiologic images to explain short-time intratumoral oxygen fluctuations
Source: PLoS Comput Biol. 2021 Jul 26;17(7):e1009206. doi: 10.1371/journal.pcbi.1009206 (PMC8341701; doi:10.1371/journal.pcbi.1009206)
Supplement: Attachment — Submitted filename: Kingsley_PCB_refMarch2021.pdf [file pcbi.1009206.s002.pdf]

Title: Bridging cell-scale simulations and radiologic images to explain short-time Intratumoral oxygen fluctuations
Authors: JL Kingsley, JR Costello, N Raghunand and KA Rejniak
Your ref: **PCOMPBIOL-D-21-00412**

Summary
This is an interesting and well-written paper. The authors use an existing hybrid agent-based model to compare two mechanisms which many explain experimentally observed temporal fluctuations in tumor tissue oxygen levels. The authors combine a range of different techniques (eg parameter sensitivity analysis of their hybrid ABM, optimisation to determine time courses for the oxygen supply and uptake rates that are consistent with the data) in order to generate their results. Based on these results, they conclude that temporal fluctuations in the supply of oxygen from the vasculature are more likely to explain the data than temporal fluctuations in the uptake of oxygen by tumor cells. These results and conclusions are interesting and represent a welcome contribution to the literature.

While I am supportive of publication of the article, I would first recommend that the authors revise their article paying particular attention to the following: (i) the limitations of their model, (ii) its relationship to existing models in the literature (ie include a review of the relevant modelling literature, including alternative approaches that could be used to analyse the experimental data), and (iii) more details about the data which motivates their study.

I include below more detailed comments.

Comments
- [**Abstract, line 44-46**] It would be great if the authors could include some preliminary results comparing the responses to treatment of different tissue morphologies that are compatible with a particular dataset (or ROI).
- [**Discussion of Figure 1**] Please clarify the difference between the data in Figures 1B and C. In particular, does Figure 1C show the changes in the oxygen tension rather than the absolute values? What are the length scales in Figs 1A and B? Please also clarify what is meant by 'tissue permeability' (oxygen transport in the extravascular space?) and why these data suggest that the observed fluctuations are not related to changes in tissue permeability (is tissue permeability distinct from vascular permeability?).
- **[Section 1. Introduction]** Please review the relevant modelling literature and explain how your hybrid model relates to existing models. For example, there are many more detailed models that account for blood flow in 2D and 3D. Alternatively, one might consider a simpler, compartmental model which distinguishes vascular, tumor and stromal tissue compartments (see also comments below).
- **[Section 2. Methods – Mathematical Model]** Could the authors make their code available for readers, via eg GitHub repository?
- **Line 181:** what is the biological justification for assuming temporal variation in the rate at which the cancer cells consume oxygen (and not in the stromal cells)? What mechanisms might be at play here? Further, could the authors clarify how the stromal and tumor cells differ when delta_v and delta_T are constant? Both cell populations consume oxygen at the same rates when delta_T = 1? Further, is it reasonable to assume identical oxygen concentrations in each blood vessel (I think that is what you assume; if not, please clarify how they are specified)?
- **[Section 3.** Results – eg lines 200, 226] Please clarify what is meant by 'stable levels of oxygenation.' Does this refer to steady state solutions?
- [Figure 4] Would it be possible to include slices though Figure 4A. Please also clarify whether these results are for individual realisations or averages from multiple realisations.

- Line 281: can you comment on why the time to reach a steady state increases as the density of cells in the domain decreases?
- Line 284: are 25 realisations sufficient to generate meaningful statistics? Please comment.
- [Section 3.3] you perform a parameter sensitivity analysis in which you vary the densities of vessels, tumor cells and stromal cells only. Did you consider performing a global parameter sensitivity analysis?
- [Section 3.4 and Section 4. Discussion] The idea of identifying the time-dependent sequences for delta_V(t) and/or delta_T(t) that provide fits to the data is a great one. Could the authors comment on the robustness of these optimal time series for tissues with the same vascular, tumor and stromal proportions and different morphologies? It would be helpful it the authors included the fitted profiles for delta_V(t) and delta_T(t) in Figure 5. Also, do the authors have any estimates of the noise in the experimental data? Did they attempt any purely synthetic studies (+/- noise) in order to test their ability accurately to infer these time series? Did the authors attempt to fit the data to a simpler 3-compartment model (separate compartments for vasculature, tumor and stromal cells)? What extra insight is gained from using an ABM?
- **[Discussion]** please comment on how the results could be generalised for 3D tissues.

Minor Comments

- [Abstract and Introduction] It might be more natural to write the abstract and introduction in the present tense rather than the past tense (eg "Here, we provide a link … Using hybrid agent-based, we generate …") since you have not yet presented this work at this stage of the paper.
- Line 58: 'To examine the POSSIBLE causes of …' (ie there could be other causes of rapid oxygen fluctuations)
- Line 66: Replace 'a set of tissues representative of radiology images' with 'a set of tissues that are compatible with radiology images'.
- Lines 77-83 and 88: please include typical values of oxygen levels associated with chronic hypoxia and the amplitudes in oxygen fluctuations seen in cycling hypoxia.
- Line 99: define EPR
- Line 101: 'indicates' → 'suggests'?
- Line 110: Please clarify that the mechanisms you consider is not an exhaustive list and perhaps include some alternatives.
- Line 118: please clarify the sense in which the selected tissues 'match' the experimental data.
- **[Section 2. Methods – Mathematical Model]** Please clarify whether you plot oxygen distribution or oxygen gradient (same comment applies throughout the text).
- **Figure 2, legend:** please revise legend to clarify that figures depict output from a model simulation, and indicate whether results are plotted in terms of dimensional or dimensionless variables. And refer to Table 1 for parameter values.
- **Line 149:** is 'F' a fixed parameter? Further is the magnitude of the force between the different cell types and the vessels assumed to be the same in all cases? Is it reasonable to assume that cells and blood vessels exert similar forces on other cells?
- **Line 164:** what is the justification for assuming that the vessels are not subject to relocation? Have the authors considered whether the cells might exert compressive forces that occlude the vessels? Additionally, for the timescale of interest (eg 30mins), how much cell movement is anticipated/observed? Are the results significantly affected if all cell movement is neglected?
- **Table 1:** what is the scaling parameter? Where does it appear in the model?
- **Figure 3:** please clarify that this is just one realisation with particular proportions of vasculature, tumor and stromal cells.
