# Supplementary material for: Bridging cell-scale simulations and radiologic images to explain short-time intratumoral oxygen fluctuations
Source: PLoS Comput Biol. 2021 Jul 26;17(7):e1009206. doi: 10.1371/journal.pcbi.1009206 (PMC8341701; doi:10.1371/journal.pcbi.1009206)
Supplement: Attachment — Submitted filename: Response_to_reviewers.pdf [file pcbi.1009206.s003.pdf]

Professor Maini, Associate Editor of PLOS Computation Biology
Professor Mac Gabhann, Editor-in-Chief PLOS Computation Biology

We would like to thank the Reviewers for their constructive comments and suggestions that have helped us to significantly improve our manuscript. We have addressed all their comments and our changes are highlighted in red in the revised manuscript. Below, we also respond to the reviewers' comments directly.

Reviewer #1:

Summary
This is an interesting and well-written paper. The authors use an existing hybrid agent-based model to compare two mechanisms which many explain experimentally observed temporal fluctuations in tumor tissue oxygen levels. The authors combine a range of different techniques (eg parameter sensitivity analysis of their hybrid ABM, optimisation to determine time courses for the oxygen supply and uptake rates that are consistent with the data) in order to generate their results. Based on these results, they conclude that temporal fluctuations in the supply of oxygen from the vasculature are more likely to explain the data than temporal fluctuations in the uptake of oxygen by tumor cells. These results and conclusions are interesting and represent a welcome contribution to the literature.

While I am supportive of publication of the article, I would first recommend that the authors revise their article paying particular attention to the following: (i) the limitations of their model, (ii) its relationship to existing models in the literature (ie include a review of the relevant modelling literature, including alternative approaches that could be used to analyse the experimental data), and (iii) more details about the data which motivates their study.

> *Response:*
> *(i) the limitations of their model,*
> *We appreciate this suggestion. We added description of model limitations and how they can be overcome in future research in the Discussion section, paragraph 4th.*
>
> *(ii) its relationship to existing models in the literature (ie include a review of the relevant modelling literature, including alternative approaches that could be used to analyse the experimental data),*
> *We thank the reviewer for this comment. We included a review of other mathematical models relevant to the topic of our paper in the Discussion section, paragraph 5.*
>
> *(iii) more details about the data which motivates their study.*
> *We followed this suggestion and added more information about the experimental technique (EPR) and the collected data. This is now included in the Introduction section, paragraph 2.*

I include below more detailed comments.
Comments
1. [**Abstract, line 44-46**] It would be great if the authors could include some preliminary results comparing the responses to treatment of different tissue morphologies that are compatible with a particular dataset (or ROI).
   Response:
   We appreciate the reviewer's comment and, in fact, modeling treatment response is our long-term goal of this project. We added more information about this goal in the Discussion section, last paragraph. However, due to a large number of distict anti-cancer treatments, it is pre-mature at this moment to include treatment protocols in this paper. Our goal is first to show that our model is capable of reproducing experimentally observable data, i.e., changes in tissue oxygenation, before we start modeling changes due to anticancer treatment.

2. [**Discussion of Figure 1**] Please clarify the difference between the data in Figures 1B and C. In particular, does Figure 1C show the changes in the oxygen tension rather than the absolute values? What are the length scales in Figs 1A and B? Please also clarify what is meant by 'tissue permeability' (oxygen transport in the extravascular space?) and why these data suggest that the observed fluctuations are not related to changes in tissue permeability (is tissue permeability distinct from vascular permeability?).

   Response:

   We thank the reviewer for this comment that allows us to present our motivation more clearly. We updated the discussion section for Figure 1 to provide more information about the link between Figure 1B and Figure 1C. We indicated now clearly that all experimental data relates to the partial pressure of oxygen (pO_2). We appreciate the reviewer's question about the length scales in Figure 1A and Figure 1B. Unfortunately, there is no spatial scale provided in the paper [Yasui et al.] that we used as a source of our data. The phrase 'tissue permeability' was used to describe the ability of oxygen to penetrate the tissue and is related to interstitial transport. Since it was ambiguous, we removed this phrase and used 'interstitial transport' instead in the whole manuscript.

3. **[Section 1. Introduction]** Please review the relevant modelling literature and explain how your hybrid model relates to existing models. For example, there are many more detailed models that account for blood flow in 2D and 3D. Alternatively, one might consider a simpler, compartmental model which distinguishes vascular, tumor and stromal tissue compartments (see also comments below).

   Response:

   We included a review of other mathematical models relevant to the topic of our paper in the Discussion section, paragraph 5.

4. **[Section 2. Methods – Mathematical Model]** Could the authors make their code available for readers, via eg GitHub repository?

   Response:

   The code is now available at GitHub: https://github.com/rejniaklab/MultiCell-O2-fluctuations

5. **Line 181:** what is the biological justification for assuming temporal variation in the rate at which the cancer cells consume oxygen (and not in the stromal cells)? What mechanisms might be at play here? Further, could the authors clarify how the stromal and tumor cells differ when delta_v and delta_T are constant? Both cell populations consume oxygen at the same rates when delta_T = 1? Further, is it reasonable to assume identical oxygen concentrations in each blood vessel (I think that is what you assume; if not, please clarify how they are specified)?

   Response:

   We thank the reviewer for bringing to our attention that these aspects were not written clearly enough. Changes in oxygen uptake by cancer cells are often observed when the cells are grown in different microenvironmental conditions. Our own group used the Seahorse technology to measure changes in oxygen consumption in tumor cells exposed to various concentrations of pyruvate (Wojtkowiak et al. Pubmed PMID: 25635223). We added this information in section 2.2. While these processes were investigated extensively in tumor cells, there is not enough evidence of whether stromal cells (T cell, macrophages, fibroblasts, adiopocytes, dendritic cells etc.) undergo the same metabolic changes, thus we only included the metabolic changes in tumor cells.

   Tumor and stromal cells differ in size, thus even for delta_T=1, the two cell types absorb different amounts of oxygen—absorption is defined using the indicator function, thus the number of grid points in numerical implementation for both cell types is different. Our assumption that all vessels are of the same size and that the level of oxygen in each vessel is the same is simplification. This was done to reduce model complexity, since changes in tissue morphology have already introduced a fair amount of heterogeneity. Similarly, all tumor cells have the same size, and all stromal cells have identical sizes. In future research, we will release this assumption. This is now discussed in the Discussion section, paragraph 4.

6. **[Section 3.** Results – eg lines 200, 226] Please clarify what is meant by ‘stable levels of oxygenation.’ Does this refer to steady state solutions?

   Response:

   We thank the reviewer for bringing to our attention that this aspect of our model was not clearly defined. For each in silico tissue, we determined a numerically stable oxygen distribution, stable in the sense that L2-norm between two consecutive oxygen distributions is smaller than 10^{-10}. We now replaced the phrase “stable levels of oxygenation” with “a numerically stable oxygen distribution” throughout the text to make these statements more clear.

7. [Figure 4] Would it be possible to include slices though Figure 4A. Please also clarify whether these results are for individual realisations or averages from multiple realisations.

   Response:

   We examined different ways to visualize this tissue classification. We found that the 3D convex hulls from Figure 4 are the best to show stabilization levels for over 1,500 tissues. For comparison, we present below examples of individual slices (four out of ten levels for vascularity). We added in caption to Figure 4 that this graph was created with one morphology for each of the 1,530 tissue characteristics.

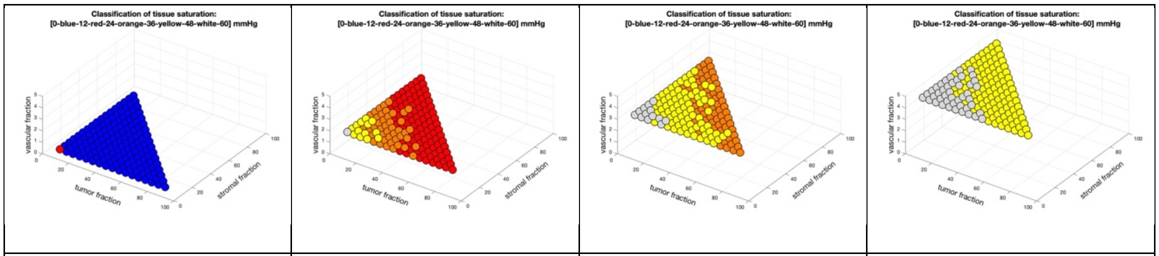

vascularity=0.5%	2%	3,5%	5%

8. Line 281: can you comment on why the time to reach a steady state increases as the density of cells in the domain decreases?

   Response:

   For the tissues with smaller number of cells, it takes longer, on average, before oxygen reaches these cells. Thus, for a longer time, oxygen spatial profile is affected mostly by its diffusion from vessels before cell absorption starts contributing to the oxygen influx-outflux balance. This information is added to section 3.2.

9. Line 284: are 25 realisations sufficient to generate meaningful statistics? Please comment.

   Response:

   We generated additions 75 tissues of the same characteristics as in Figure 4C, and the average level of oxygen at which they have stabilized was 29.79 +/- 2.5 mmHg in comparison to originally reported 29.98 +/- 2.4 mmHg. Thus, we believe that statistics from 25 realizations are sufficient.

10. [Section 3.3] you perform a parameter sensitivity analysis in which you vary the densities of vessels, tumor cells and stromal cells only. Did you consider performing a global parameter sensitivity analysis?

    Response:

    We have presented the outcomes of global sensitivity analysis in Figure 6B for both influx (green) and uptake (grey) schedules, and showed that as long as the selected tissues (with varied characteristics) have stabilized oxygen levels within +/-1.5 mmHg from the experimentally measured value, our model produces robust responses.

11. [Section 3.4 and Section 4. Discussion] The idea of identifying the time-dependent sequences for delta_V(t) and/or delta_T(t) that provide fits to the data is a great one. Could the authors comment on the robustness of these optimal time series for tissues with the same vascular, tumor and stromal proportions and different morphologies? It would be helpful it the authors included the fitted profiles for delta_V(t) and delta_T(t) in Figure 5. Also, do the authors have any estimates of the noise in the

experimental data? Did they attempt any purely synthetic studies (+/- noise) in order to test their ability accurately to infer these time series? Did the authors attempt to fit the data to a simpler 3-compartment model (separate compartments for vasculature, tumor and stromal cells)? What extra insight is gained from using an ABM?

Response:

Following the reviewer's suggestion, we performed additional simulations keeping tissue characteristics fixed, but changing tissue morpohologies (50 tissues for each optimal schedule). The obtained results showed similar robustness as presented in Table 3 for tissues of different characteristics but similar stabilized oxygen level. Here, among tissues with stabilized oxygen level within 3.5 mmHg of the experimental data, the optimal influx schedule fitted the experimental results with L_2-norms below 0.2 for the following number of cases: ROI#1: 15 out of 46; ROI#2: 18 out of 48; ROI#3: 9 out of 48; ROI#4: 41 out of 41. Similarly, as in Table 3, the optimal uptake schedule performed worse than influx schedule, and the following number of cases fitted the experimental results with L_2-norms below 0.2: ROI#1: 0 out of 46; ROI#2: 14 out of 48; ROI#3: 1 out of 28; ROI#4: 4 out of 41 (though 26 out of 41 has L_2-norm below 0.25). All successful cases have stabilized oxygen level within 1.5 mmHg of the experimental data showing that several different morphologies performed well if the level of stabilized oxygenation was close to the experimental data. We added more details about the case presented in Supporting Information S5. The fitted profiles are shown in Figure 4 for both influx (blue triangles) and uptake (red stars) schedules. All values of delta_V(t) and delta_T(t) are listed in Table 2. The experimental data was recorded from in vivo experiments and only data from the four indicated ROIs was included in experimental publication, and was available to us. Moreover, the fluctuation profiles are specific to a given ROI, thus no replicates were available. We are not aware of any synthetic studies done by this experimental group. Our goal was to construct a model to match data from the four ROIs. The ABM models provide a way to reconstruct tissue morphologies which is impossible with compartmental models that assume well-mixing of all model components.

12. **[Discussion]** please comment on how the results could be generalised for 3D tissues.

    Response:

    We thank the reviewer for this suggestion. We added description of model extension to the 3D space in the Discussion section, paragraph 4.

Minor Comments

13. [Abstract and Introduction] It might be more natural to write the abstract and introduction in the present tense rather than the past tense (eg "Here, we provide a link ... Using hybrid agent- based, we generate ...") since you have not yet presented this work at this stage of the paper.

    Response:

    We followed the Reviewer's suggestion and rewritten the abstract and introduction.

14. Line 58: 'To examine the POSSIBLE causes of ...' (ie there could be other causes of rapid oxygen fluctuations)

    Response:

    This was corrected

15. Line 66: Replace 'a set of tissues representative of radiology images' with 'a set of tissues that are compatible with radiology images'.

    Response:

    This was corrected

16. Lines 77-83 and 88: please include typical values of oxygen levels associated with chronic hypoxia and the amplitudes in oxygen fluctuations seen in cycling hypoxia.

    Response:

    This information was added

17. Line 99: define EPR

    Response:

    This was corrected by describing the EPR imaging technique in the Introduction section

18. Line 101: 'indicates' -> 'suggests'?

    Response:

    This was corrected

19. Line 110: Please clarify that the mechanisms you consider is not an exhaustive list and perhaps include some alternatives.

    Response:

    In general, the distribution of oxygen within the tissue depends on the amount and localization of oxygen entering the tissue, on the amount and localization of oxygen that leaves the tissue and its transport through the tissue. We are addressing the first and second processes here. The changes in interstitial transport can be due to the change in the number or localization of cells and vessels, and due to changes in the composition of the extracellular matrix. Since we consider very short period of time (30 minutes) and do not include any therapeutic interventions (such as radiotherapy or surgery), there is no change in the number of cells (no proliferation, no death), no changes in the vasculature (no angiogenesis, no vascular collapse due to tumor growth), and no changes in the ECM composition (no ECM production by stromal cells). Therefore, the only processes are: source term of oxygen—vascular influx and sink term of oxygen—cellular uptake. We do not see any other naturally appearing mechanisms. We explained that better in Introduction, paragraph 3.

20. Line 118: please clarify the sense in which the selected tissues 'match' the experimental data.

    Response:

    The tissues were selected to match the average pO_2 value from each experimental ROI. This is now clarified.

21. **[Section 2. Methods – Mathematical Model]** Please clarify whether you plot oxygen distribution or oxygen gradient (same comment applies throughout the text).

    Response:

    We thank the reviewers for pointing out this issue. We used the term 'gradient' in a more colloquial way not mathematical. However, indeed, all plots show oxygen distribution. This is now corrected throughout the text.

22. **Figure 2, legend:** please revise legend to clarify that figures depict output from a model simulation, and indicate whether results are plotted in terms of dimensional or dimensionless variables. And refer to Table 1 for parameter values.

    Response:

    This is now corrected

23. **Line 149:** is 'F' a fixed parameter? Further is the magnitude of the force between the different cell types and the vessels assumed to be the same in all cases? Is it reasonable to assume that cells and blood vessels exert similar forces on other cells?

    Response:

    The repulsive forces are used only in the initial phase of the simulation to define tissue morphology, and with a goal to resolve overlapping between cells and vessels. This use of repulsive forces is illustrated in Supportive Information S1. Once the tissue is created, the repulsive forces are deactivated, since the cells and vessels are immobile during the simulations of oxygen fluctuations. The same spring stiffness is used for all repulsive forces to resolve the overlapping conditions. We updated section 2.1 to presented more details of this process.

24. **Line 164:** what is the justification for assuming that the vessels are not subject to relocation? Have the authors considered whether the cells might exert compressive forces that occlude the vessels? Additionally, for the timescale of interest (eg 30mins), how much cell movement is anticipated/observed? Are the results significantly affected if all cell movement is neglected?

Response:
During the considered time period (30 minutes) there is no significant proliferation in in vivo tumors, thus there are no new compressive forces that could lead to vessel collapse. All vessels in our simulations are considered functions. Tumor cells in tissues are less mobile than in in vitro experiments, since both ECM and other cells form migration barriers. In in vitro experiments cells move a fraction of micron per minute, thus we are confident that cell migration can be neglected in our simulations.

25. **Table 1:** what is the scaling parameter? Where does it appear in the model?
Response:
We thank the reviewer to point out this issue, so we can present it more clearly. The scaling parameter is used to scale mass units for all variables related to oxygen (oxygen uptake rates, Michaelis constants, vascular oxygen level, all listed in Table 1). The unit scaling is used to achieve better numerical stability, that is to avoid using values that differ in orders of magnitude. We introduced the "sigma" unit since there is no officially named unit for 10^{-19}. Thus, sigma-gram=0.05 atto-gram. We updated this information in Table 1.

26. **Figure 3:** please clarify that this is just one realisation with particular proportions of vasculature, tumor and stromal cells.
Response:
This is now corrected

Reviewer #2:
The manuscript by Kingsley et al. presents a computational investigation into the cause of small-scale oxygen fluctuations in solid tumours. An agent-based model is used to generate a set of micro-scale tissue morphologies which emulate voxel-scale O2 gradients observed in squamous cell carcinoma VII (SCCVII) in vivo, using electron paramagnetic resonance imaging (EPRI; data from Yasui et al. (2010)). Using this framework, the authors showed that changes in intravascular O2 reproduce the magnitude of fluctuations in vivo whereas modifying cellular absorption did not.

The written language of the manuscript was at times fragmented which hindered readability but I enjoyed the novelty of generating an agent-based modelling framework to infer plausible explanations of in vivo observations. That said, I have significant reservations regarding the model's application to EPRI data which may impact the biological conclusions of the manuscript.

(i) In the Supplementary Material the authors state that the "model represents features characteristic of many solid tumors, thus we did not focus on any specific tumor type". I agree. The modelling framework can, to an extent, represent certain features of solid tumours. However, not focusing on tumour type in this manuscript when attempting to recreate pO_2 fluctuations observed by Yasui et al. (2010) (Fig. 1 in Kingsley et al.) in SCVII tumours, I believe, is an error. Tumour microenvironment heterogeneity can vary wildly between tumour types and so by not targeting SCVII tumours may negate the authors' biological inferences.
Response:
We thank you the reviewer for this comment. We agree that tumor architecture and the microenvironment can vary significantly between cancers of different types. However, the fast oxygen fluctuations were observed in several different solid tumors. Yasui et al. reported pO2 fluctuations in both mouse squamous carcinoma and in human colorectal carcinoma; Christodoulou et al. (PMID: 27498337) reported such changes in fibrosarcomas; and Khan et al. (PMID: 22079559) in gliomas. Therefore, this phenomenon occurs in multiple tumors with different microenvironments. We used experiments described in Yasui et al. as motivation for our more general studies. This is now indicated in the main text.

(ii) To mimic the fluctuations and magnitudes in O2 observed by Yasui et al. (2010) in silico, the authors should (where possible) parameterise their models with respect to SCCVII tumours. For example, Kingsley et al. used a constant vessel diameter of 40 microns, yet SCVII have diameters in the range of 14.5 – 11.0 microns for tumours of similar masses [1] (see Fig. 23 in [1]). The authors here also increment tissue vascularity from 0.5 to 5%. However, Yasui et al. (2010) reports vascular density of 27.7 ± 2.1% and 8.1 ± 1.0% for small and large SCVII tumours, respectively. Similarly, [1] reports vascular density as between 2 – 7.5% for large SCVII tumours (see Fig. 25 in [1]). Generating new tissue morphologies with these reported values may not change the authors' conclusion regarding cycling hypoxia but will clearly alter the range of morphologies which replicate experimentally observed pO2 fluctuations.

Response:

We thank the reviewer for this comment and for pointing out to an additional source material. Yasui et al. indeed reported on blood volume (%) in Figure 4 and related this to blood vessel density of 27.7 ± 2.1%. They also reported on vascular density of 3.6 ± 0.2% as determined from tumor histology with CD31 staining, Figure 5. Since blood volume is determined from radiologic images on a voxel scale (average value), while the CD31 staining relates directly to tissue morphology, we used these data for our morphology-based computer simulations. In Figure 5 the vessel density as a % of the optical field is reported as <4%. Therefore, we used the values between 0.5% and 5% in our simulations. The decision to use vessels of a uniform size was intentional to simplify the model because we already varied several other model aspects. For the same reason all cells are of the same size. In our future research we will incorporate more heterogeneities in the tissue. This is now indicated in the Discussion.

(iii) In general, the manuscript would benefit from relating its conclusions back to the study of Yasui et al. (2010) in terms of the additional insights the in silico model provides. For example, Kingsley et al. indicate that upstream effects (via varying vessel influx levels) replicate O2 gradients observed in the EPRI data. Comparatively, using analysis of the SCVII tumours via immunohistochemistry, Yasui et al. (2010), pO2 fluctuations correlated with pericyte density (i.e., local vascular functionality) rather than vascular density. Kingsley et al. could use their model to indict whether this hypothesis may be true, whilst highlighting that it does not currently include local vascular functionality but has the scope to in the future.

Response:

We thank the reviewer for this comment. We followed this suggestion and relate our conclusions back to the Yasui et al. study. This is now updated in the two first paragraphs of the Discussion section. At the current stage our model is not dealing with local vascular functionality, but we will consider this in the future. This is now included in the Discussion section in paragraph 4, when the future model directions are listed.

(iv) Presenting the generalised modelling framework and then applying it to the EPRI data to make conclusions regarding SCVII tumours I believe would really elevate the manuscript.

Response:

Our approach was to use the Yasui et al. results as motivation for our model general studies and not focusing on the SCCVII tumor exclusively. Therefore, we respectfully disagree with the reviewer, and decided to keep the current format of our manuscript.

The following are further points which could improve the manuscript:

1. There are several places in the manuscript where I would expect a reference to support a written statement but one is not given. For example, Line 76 regarding oxygen gradients at 120-180 um from the vasculature.

Response:

This is now corrected

2. The introduction dives straight into discussing the ROIs observed in Yasui et al. (2010). However, a brief explanation of how EPRI works along with spatial information such as EPRI resolution in comparison to microenvironment features in the authors' tissue morphologies. This would develop the reader's understanding from the start of the limitations of EPRI and how the manuscript seeks to link the micro- and macro-scales.

Response:
We thank the reviewer for this comment that allowed us to improve description of experimental data that motivated our work. We now included more information about the EPR technique and data in the Introductions section, as well as information about scale differences between EPR and our mathematical model.

3. Lines 126 and 491, voxel dimensions are defined as mm2 – voxels are volumetric.
Response:
The reviewer is right that voxels from radiologic images are volumetric. We corrected that in Introduction and also indicated that our 2D mathematical model corresponds to the voxel cross section.

4. Following (3), the authors do not discuss the fact that the model is 2-dimensional (although not explicitly stated) yet EPRI is 3-dimensional. From Yasui et al. (2010), I assume that the ROIs from are 3-dimensional ROIs and that plot of changes in pO2 (Fig 2c) is an average for a given ROI? This should be stated and highlighted that the current model does not incorporate 3D spatial heterogeneities.
Response:
The reviewer is right. We corrected that in Introduction. We also indicated that our mathematical model is 2D and corresponds to the voxel cross section. We included in the Discussion section information that current model does not incorporate 3D spatial heterogeneities and that adaptation to full 3D model will be considered in our future research.

5. The authors discuss “large” fluctuations in tumour oxygenation. It would be useful to write in-text what the authors consider as large (i.e., magnitude).
Response:
Based on experimental data, in two ROIs the pO_2 fluctuation are relatively small, not exceeding 2-3 folds, and in two other ROIs the fluctuations are larger, more than 5-fold of change. This is now explained in Introduction and in section 3.4.

6. Line 165 to 166 state that vessels can overlap to represent shapes observed in histological slices and that further information of the algorithm is provided in Supporting Information S1. However, the only indication of overlapping vessels is in Fig S3f. Further, examples of overlapping vessels with similar images of these shapes in histological images would be useful. In addition, where vessels with multiple overlaps as in Fig Sf (see 3 overlapping vessels in the bottom left-hand corner) excluded from the library of tissue samples? I imagine these shapes are not observed in histology and so should be omitted.
Response:
We thank the reviewer for this comment. We updated Supporting Information S1 to include tissue patch with overlapping vessels. The use of multiple overlapping vessels is a simple approximation of more complex vessel shapes which are often seen in histology images (example below). They are the result of an oblique cut, while the circular vessels show cross section cut perpendicularly.

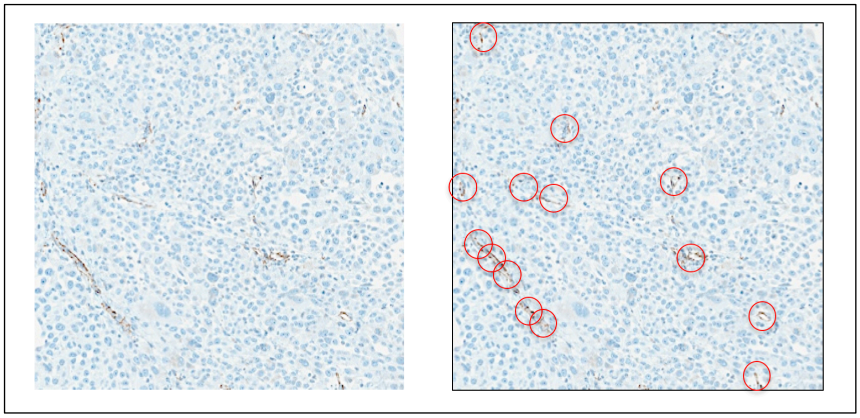

7. Lines 507 to 534 in the Discussion reads like a literature review of imaging methods to observe tissue oxygenation. It is odd that this forms a significant portion of the Discussion.

Response:

We thank you the reviewer for this comment. Our goal was to present how other radiologic images can provide other data to improve future model predictions. Apparently, we did not make good enough job. This part of our manuscript is now shortened and rewritten.

8. A discussion of the model limitations of the model / future development (heterogeneous vasculature and O2 influx, 3D etc) the Discussion would be very useful.

Response:

We thank the reviewer for this suggestion. We added description of model limitations and how they can be overcome in future research in the Discussion section, paragraph 4.

9. It would be advisable to use colour blind safe colour schemes for the figures.

Response:

We respectfully disagree with change of color schemes. The colors used for oxygen distribution corresponds to color scheme of EPRI (cyan-low pO_2 level, white-high pO_2 level). The color scheme used for tissue morphology corresponds to typical colors in histology images, and H&E staining (pink, purple and red-brown colors). This explanation is now included in the Figure 2 caption.

10. In Fig 4., the iteration subfigures on the right-hand side may be presented better on a log x-axis.

Response:

We examined all figures showing evolution and numerical stability of the average tissue oxygenation, but we feel that the current graphs better present the stability concept. Thus, we decided to keep the current graphs.

11. In Fig 5., the subfigures showing oxygen vs. time should be rescale to 0 – 40 mmHg and given a clear colour scheme. Currently, it is difficult to see the data points.

Response:

We thank the reviewer for this comment. The panels in Figure 5 have been rescaled, and we used more visible markers to show computational results.

12. Supplementary Figure S5 is far too small to read. Consider splitting and enlarging into several figure panels.

Response:

We followed reviewer's suggestion and have split this figure between two pages.
