# Supplementary material for: Bridging cell-scale simulations and radiologic images to explain short-time intratumoral oxygen fluctuations
Source: PLoS Comput Biol. 2021 Jul 26;17(7):e1009206. doi: 10.1371/journal.pcbi.1009206 (PMC8341701; doi:10.1371/journal.pcbi.1009206)
Supplement: Attachment — Submitted filename: Response to Reviewers2.docx [file pcbi.1009206.s004.docx]

*Professor Maini, Associate Editor of PLOS Computation Biology*

*Professor Mac Gabhann, Editor-in-Chief PLOS Computation Biology*

*We have addressed all Reviewers’ comments and our detailed responses are listed below. Moreover, all our changes are highlighted in red in the revised manuscript.*

Reviewer #1:

I have read through the revised manuscript and the authors response to the comments I raised. Overall, the authors have done a good job of addressing most of the comments that were raised. However, there are a few places where further clarification is needed. I will list these below.

Comment 2. The authors justify their decision not to include dimensional estimates of the length scales involved in their model by stating that there is no spatial scale provided in the experimental paper on which there work is based. I do not consider this sufficient justification – the authors could, for example, estimate typical length scales either from the existing experimental literature or length scales consistent with the vascular densities that they use in their simulations. I would encourage them to do this, so that the reader more readily understands the physical situations that they are modelling.

Response:

We never refused to state the dimensions of our model. All figures showing our spatial simulations (Figures 2-5) include domain dimensions: 1000x1000μm^2^. We also stated in lines 157-159 that “we consider a two-dimensional tissue patch with an area of 1 mm2 that corresponds to a cross section of a typical EPR imaging voxel and a single element in the EPR oxygen map”. Now, we also added this information to Table 1 for clarity.

The Reviewer’s original comment refers to experimental not computational data and reads: *“What are the length scales in Figs 1A and B?“* In response, we honestly answered that “*We appreciate the reviewer’s question about the length scales in Figure 1A and Figure 1B. Unfortunately, there is no spatial scale provided in the paper [Yasui et al.] that we used as a source of our data.”* It would be unethical to impose our estimates on the data that did not come from our lab. However, Yasui et al. provided the size of the whole tumor (>1,200mm^3^) that we used for quantification and this information is now stated in line 104 and in caption of Figure 1.

Comment 3. The literature review in the discussion does not reflect the range of modelling approaches in the literature. The authors may find the following references of relevance:
• T Roque, L Risser, V Kersemans, S Smart, D Allen, P Kinchesh, S Gilchrist, AL Gomes, JA Schnabel, MA Chappell (2016). [1]Reaction-Diffusion Model of Solid Tumour Growth. IEEE Trans Medical Imaging (DOI:10.1109/TMI.2017.2779811)
• C Villa, MAJ Chaplain & T Lorenzi (2021). Modeling the emergence of phenotypic heterogeneity in vascularized tumors. SIAM J Applied Mathematics, 81(2), 434-453.

Response:

These references have been added: #45 and #46

Comment 5: please can the authors clarify the relevance of measured changes in pyruvate levels obtained from in vitro experiments. I do not understand how changes in oxygen consumption in response to changes in pyruvate levels provide information about how cells response to changes in oxygen levels. Equally, I respectfully disagree that the impact of changes in oxygen levels on stromal cells has not been studied in the literature. See for example G Solani et al (2011), B Zhao et al (2016).

Response:

The original Reviewer’s comment was: *“what is the biological justification for assuming temporal variation in the rate at which the cancer cells consume oxygen (and not in the stromal cells)? What mechanisms might be at play here?”* We gave an example that was performed in our group [Wojtkowiak … Rejniak … Gillies, Cancer Metabolism 2015, Figure 2] in which the same cancer cells showed variations in the rate at which oxygen was consumed because the cells were exposed to different levels of pyruvate. This observation was our motivation to model variation in the rate at which cancer cells consume oxygen. In the current paper, we examined how high such variations in oxygen consumption needed to be to reproduce the experimentally observable oxygen fluctuations in the tissue patch. However, for simplicity, we did not specify the reason for such variations in cellular consumption of oxygen. Similarly, to reduce the number of changing components, we only considered oxygen consumption by cancer cells. In our future work, we will incorporate different stromal cells (fibroblasts, T cells, B cells, MDSC cells, Natural killer cells, dendritic cells, macrophages, adipocytes, etc.) and their heterogeneous cellular uptake. This is stated in line 572.

Comment 6: please clarify what an L2-norm of 10^{-10} means – the significance of this value will depend on the oxygen distributions being measured.

Response:

When mathematical models are solved using numerical methods, one cannot expect that the stable solution will result in the derivative being equal uniformly to 0 in the whole domain. Instead, the numerical stability is achieved if the difference between oxygen values at the consecutive iterations is very small. Our objective is to reach a situation when differences between consecutive oxygen concentrations at every grid point are very small, thus we used L_2_-norm. The value 10^-10^ was chosen since it is very small when compared to maximal oxygen value of 60mmHg at the vessel locations. Thus L_2_-norm<10^-10^ means that at every grid point the change in oxygen value between two consecutive iterations is several orders of magnitude smaller than the maximal oxygen value in the domain.

Comment 8: I do not understand why it takes longer for the system to reach a steady state as the density of cells increases. For a domain of the same size, the diffusive timescale will be the same. Does the dominant balance change in some way? The justification here could be further clarified

Response:

The diffusive time scale depends not on the domain size but on the oxygen source strength (number of vessels and oxygen influx level) and on the oxygen sink strength (number of cells and cellular uptake level). For example, when the vessel is occluded, the diffusion time and space scales will be 0 independent on the domain size. In the three cases under discussion that are showed in Figure 4B-D, we provide below additional information in the form of the contourplots showing changes in tissue oxygen levels in the low-density tissue (magenta stars) at the later iterations, but not in the higher density tissues that stabilize earlier. Here, the time to reach numerical stability with L_2_<10^-10^ vary for tissues of different cellularity, because with lower number of cells, there are more grid points located between the cells at which the oxygen content is changing due to diffusion not cellular uptake, though the changes are on a very small scale.this contributed to the larger number of iterations until the overall L_2_-condition is satisfied.

The length of oxygen profile stabilization does not play any role in the questions we want to answer. It is only used to generate the initial tissue for further simulations. When reconstructing the fluctuation patterns, we only run simulations for a number of iterations that correspond to the 3-minute intervals, when the experimental measurements are done; we do not require that the oxygen profiles at the end of each 3-minute interval is numerically stable, as it is not in the biological experiment.

If the authors can satisfactorily address the above comments, then I would be very happy to recommend their article for publication. It is a great piece of work, improved with the revisions they have made.

Reviewer #2:

In the revised manuscript, the authors have provided increased clarity and detail, particularly in the introduction and discussion, which has elevated the manuscript. I would also like to thank the authors on their detailed response particularly with regard morphological heterogeneities. Their comments have satisfied my initial concerns. I appreciate that more tumour-specific functional and architectural heterogeneities can by incorporated in future work, which has been noted in the discussion.

Subject to the minor comments below, I recommend this manuscript for publication and congratulate the authors.

- Lines 132 / 133 – the authors’ write “…too short for significant modifications in oxygen transport”. Is this comment regarding cellular update and interstitial transport?

Response:

This comment refers to the interstitial transport. This paragraph is now rewritten as follows in lines 131-133:

“We consider here the first two mechanisms only. Since the experiments were performed over a very short period of time (30 minutes), no significant modifications in oxygen transport were expected. Such modifications can arise only due to changes in the number or localization of cells or vessels, or if the extracellular matrix composition changes.”

- This reviewer apologises for their poor eyesight but Supporting Figure S4 is still too small. An alternative to reordering the figure panels may be to increase the resolution of the image in the Word Doc, as it is currently too pixelated even when zooming in.

Response:

We sincerely apologize for this issue. We have no intention to cause harm when reading our paper, and we thank the reviewer for pointing out that the resolution of Figure S5 is too low. Indeed, we did not handle these images well when reformatting the Figure. This is now corrected, and, hopefully, the new Figure S5A-D has satisfactory clarity.
